# A Roadside LiDAR and HD-Map Framework for Early Wrong-Way Driving Detection

**DOI:** 10.3390/s26216770

**Published:** 2026-10-26

**Authors:** Minsoo Kim, Pillsu Kim, Hoyeong Shin, Woosuk Sung

**Affiliations:** Department of Smart Vehicle System Engineering, Chosun University, Gwangju 61452, Republic of Korea; msmw1023@chosun.kr (M.K.);

**Keywords:** wrong-way driving, LiDAR, infrastructure-based detection, high-definition map, lane-level semantic reasoning, early warning system

## Abstract

**Highlights:**

**What are the main findings?**

- A roadside LiDAR and HD-map-based framework was developed for early wrong-way driving detection.
- The framework detected all 36 annotated WWD events, with no false-positive events observed during staged daytime and nighttime validation.

**What are the implications of the main findings?**

- The results support the feasibility of infrastructure-based LiDAR for wrong-way driving early warning.
- Lane-level semantic reasoning can improve directional decision robustness in complex road environments.

**Abstract:**

Wrong-way driving (WWD) is a rare but highly dangerous traffic event that can lead to severe head-on collisions. This study proposes a LiDAR-based infrastructure system for early WWD detection using roadside sensing and high-definition map semantics. The proposed framework integrates object detection, multi-object tracking, and lane-level directional reasoning to determine whether observed vehicle motion is consistent with the intended lane direction. To improve robustness in complex geometries, the system combines consecutive-frame validation with event-level heading analysis relative to the intended lane direction. The method was evaluated under both daytime and nighttime operating conditions using condition-specific ground-truth event annotations. In the staged daytime and nighttime validation, the framework detected all 14 daytime and 22 nighttime annotated WWD events, with no false-positive (FP) event observed among 67 daytime and 88 nighttime normal vehicle passes. The observed FP event rate was 0 events/h in both conditions, and the average time to detection was −0.36 s in daytime and −0.297 s at nighttime, indicating that the system generally identified WWD behavior before the annotated event onset. Under the evaluated staged surrogate-site conditions, these results demonstrate the feasibility of the proposed framework for event-level early WWD detection. Long-duration operation, downstream warning actuation, and validation across diverse roadway geometries and environmental conditions remain to be evaluated.

## 1. Introduction

Wrong-way driving (WWD) on divided highways is a relatively infrequent but highly severe crash type that often leads to high-speed head-on collisions. National analyses have shown that WWD continues to cause hundreds of fatalities annually in the United States. The National Transportation Safety Board reported an average of about 360 deaths per year in approximately 260 fatal wrong-way collisions, while a later AAA Foundation analysis reported 2921 fatal wrong-way crashes and 3885 deaths during 2010–2018, corresponding to an average of about 430 deaths per year [1,2]. Compared with typical freeway crashes, WWD crashes are disproportionately severe. Prior state-level studies reported fatality rates for wrong-way crashes that were roughly one order of magnitude higher than those of other crash types, and more recent state analyses continue to show markedly elevated fatal crash risk [3,4]. Exit ramps and interchange ramp terminals have repeatedly been identified as the primary entry locations for wrong-way maneuvers, while wrong-way re-entry from service, parking, or rest-area-related facilities has also been documented as a contributing scenario [1,5]. Complex interchange and ramp-terminal geometry can increase driver workload by forcing rapid interpretation of signs, pavement markings, and lane assignments within a short decision window. These risks are exacerbated under dark or visually degraded conditions, which are frequently associated with WWD events [4,5,6].

Existing WWD countermeasures span physical/geometric treatments, traditional intelligent transportation systems-based monitoring and warning systems, and more recent connected-vehicle or in-vehicle alerting approaches [3,5,7]. Vehicle-based warning approaches can be effective, but their benefits depend on vehicle equipment availability, system penetration, and the reliable operation of onboard sensing and alert interfaces [7]. Surveillance-oriented systems based on CCTV or video analytics often face practical limitations related to manual verification, calibration drift, occlusion, and false alarms, all of which can reduce the timeliness and reliability of intervention [7,8]. Physical countermeasures such as enhanced signing, pavement arrows, channelization, and geometric modifications can reduce inadvertent wrong-way entries, but their effectiveness is often site-dependent and may require substantial installation, maintenance, and location-specific design adaptation [3,5].

These limitations motivate a shift toward infrastructure-based intelligent safety systems that can monitor approaching traffic independently of vehicle onboard equipment and support early warning before a wrong-way maneuver develops into a severe conflict. Roadside infrastructure sensing is particularly attractive because it can increase information coverage for all road users, including vehicles without high-quality onboard sensing, and can help mitigate occlusion and blind-spot limitations that are difficult to resolve from a vehicle-only perspective. In this sense, infrastructure-based monitoring can complement both passive treatments and vehicle-based warnings by enabling direct observation of abnormal maneuvers at known high-risk locations [9,10].

Recent autonomous driving research has increasingly integrated behavioral decision making, trajectory prediction, path planning, and motion control to improve safety in dynamic traffic environments [11,12]. Such vehicle-side developments increase the value of reliable infrastructure-side perception at complex access locations, where roadway geometry and occlusion can complicate directional interpretation. The proposed roadside LiDAR and HD-map framework is intended to provide infrastructure-side contextual information that may complement, rather than replace, onboard autonomous driving decision and control systems.

Among roadside sensing modalities, LiDAR is attractive for safety-critical infrastructure applications because it provides high-resolution three-dimensional measurements, broad-area coverage, and relatively robust performance across daytime and nighttime conditions. These characteristics make LiDAR well suited to trajectory-level monitoring and directionality analysis in geometrically complex ramp-terminal environments [13]. More importantly, when roadside perception is integrated with high-definition (HD) maps, observed vehicle motion can be interpreted relative to explicit lane geometry, travel direction, and topological connectivity rather than raw kinematic information alone. HD maps provide lane-level semantic information such as lane boundaries, lane types, directional attributes, and legal connectivity between road segments [14]. Recent infrastructure-assisted HD mapping studies further show that vehicle trajectory density, direction, and direction variance obtained from roadside observations can support lane-level modeling and topology-aware reasoning [15]. This combination of roadside sensing and HD map semantics is therefore promising for early recognition of motion that is inconsistent with the intended roadway structure.

In this context, this study proposes a LiDAR-based infrastructure system for early WWD detection in complex roadway access environments, motivated by highway rest-area access zones and ramp-terminal settings. The proposed system integrates online object detection, multi-object tracking, short-term trajectory prediction, and lane-level semantic reasoning based on an HD map. Unlike general-purpose perception pipelines that primarily emphasize object classification or long-horizon trajectory prediction, the present system is designed to prioritize robust directionality analysis for early wrong-way recognition. By jointly evaluating lane occupancy, heading consistency, and temporal persistence across consecutive observations, the system aims to suppress transient artifacts and false alarms while enabling timely identification of genuinely hazardous wrong-way maneuvers.

The main contributions of this paper are summarized as follows:

- An infrastructure-based framework for early WWD detection in complex roadway access environments is presented using roadside LiDAR sensing and HD map semantics.
- A direction-oriented perception and reasoning pipeline is developed by combining object detection, multi-object tracking, short-term trajectory prediction, lane occupancy verification, and heading consistency analysis.
- A lane-level semantic reasoning framework is introduced to assess whether observed vehicle motion is consistent with the intended roadway structure.
- The proposed system is validated using real-world roadside sensing data under daytime and nighttime conditions, and its event-level detection performance is analyzed using temporal matching metrics.

## 2. Related Work

This section presents a concise review of prior studies related to WWD detection and prevention, with a focus on infrastructure-based perception and map-assisted behavioral analysis. Existing approaches are categorized according to sensing location and system architecture, including vehicle-based systems, surveillance-oriented monitoring, and infrastructure-side treatments. Particular attention is given to roadside LiDAR-based perception and HD map-assisted reasoning frameworks, which form the foundation of the proposed system. This review aims to clarify the positioning of the present work relative to existing studies and to highlight the gap addressed by a direction-oriented, detection-focused infrastructure system.

### 2.1. Wrong-Way Driving Countermeasures

WWD has been extensively studied due to its high fatality risk despite its relatively low occurrence frequency. Early studies and national investigations have consistently identified exit ramps and interchange ramp terminals as primary entry locations for wrong-way maneuvers, while erroneous re-entry from service, parking, or similar roadside facilities has also been documented as a contributing scenario [1,5]. These findings motivated the development of various countermeasures targeting both driver behavior and roadway design. Existing WWD countermeasures can be broadly categorized into vehicle-based systems, surveillance-oriented monitoring, and infrastructure-side physical or electronic treatments [3,5,7]. Vehicle-based warning approaches, including navigation-based alerts and onboard driver-assistance functions, depend on equipment availability, system penetration, and the reliable operation of onboard sensing and warning interfaces [7]. Surveillance-oriented approaches typically rely on CCTV or video analytics linked to traffic management centers, but in practice they often remain constrained by manual verification, calibration sensitivity, occlusion, and false alarms rather than fully automated response [7,8]. Infrastructure-side treatments such as enhanced signing, pavement arrows, channelization devices, and geometric modifications can reduce inadvertent wrong-way entries, but their effectiveness is often site-dependent and may require substantial installation, maintenance, and location-specific design adaptation [3,5]. These limitations have motivated growing interest in infrastructure-based intelligent systems capable of real-time detection and early intervention [9,10,13].

### 2.2. Infrastructure-Based Perception for Traffic Safety

Infrastructure-based perception enables roadway operators to monitor traffic behavior independently of vehicle-mounted sensing quality and can help mitigate blind spots and occlusions that are difficult to resolve from a vehicle-only perspective [9]. By embedding sensing and processing capabilities into the roadside environment, such systems can observe vehicles approaching a monitored location and support early detection of hazardous maneuvers before severe conflicts occur [9,10]. Camera-based systems can support WWD detection using existing surveillance infrastructure and provide visual information for scene interpretation [8]. However, camera performance may be affected by low illumination, weather, occlusion, calibration drift, glare, and viewing conditions [13]. Radar is also used in WWD detection systems and can provide vehicle range and speed information [10,13]. In contrast, roadside LiDAR provides three-dimensional point-cloud measurements that support vehicle localization and trajectory-level monitoring [13]. In the present study, LiDAR was selected because these spatial measurements can be integrated with lanelet geometry for lane-level directional reasoning under the evaluated daytime and nighttime conditions. This task-specific choice does not imply that LiDAR is universally superior to camera- or radar-based sensing; multimodal sensing remains a relevant direction for future deployment.

### 2.3. LiDAR-Based Object Detection and Tracking in Infrastructure Settings

Roadside LiDAR has gained increasing attention for traffic monitoring and safety applications because it provides high-resolution three-dimensional measurements and broad-area spatial coverage [13]. Its ability to operate independently of ambient illumination also makes it suitable for infrastructure-based monitoring under daytime and nighttime conditions [16]. Zhang et al. demonstrated roadside LiDAR-based vehicle tracking and speed estimation, showing that roadside point-cloud observations can be used to reconstruct vehicle trajectories for traffic analysis [17]. Zhang and Jin developed a detection-and-tracking framework based on range and intensity background subtraction, demonstrating the extraction of vehicle trajectories from roadside LiDAR observations [16]. More recently, Zhang et al. proposed an anchor-free real-time tracking approach for generating microscopic vehicle trajectories from roadside LiDAR data [18]. Roadside LiDAR performance also depends on installation configuration, particularly sensor placement and the resulting coverage and blind zones. Ge et al. examined the effects of roadside LiDAR configuration on vehicle detection and tracking performance [19]. Luo et al. showed that full occlusion and limited point returns can fragment vehicle trajectories and destabilize estimated vehicle position and speed, motivating explicit treatment of perception uncertainty in downstream traffic-behavior analysis [20]. These studies establish roadside LiDAR as a useful basis for vehicle detection, tracking, and trajectory reconstruction. However, their primary emphasis is generally on perception accuracy, trajectory quality, or traffic monitoring rather than on event-level early recognition of WWD. The present study addresses this distinct problem by combining roadside LiDAR perception, map-based predicted paths, and lanelet-based semantic reasoning to evaluate whether vehicle motion is consistent with the intended direction of the associated lanelet.

### 2.4. HD Map-Based Behavioral Reasoning

HD maps provide lane-level geometric and semantic information, including lane directions, connectivity, and permissible driving maneuvers [14]. Recent studies have demonstrated that HD maps can significantly enhance infrastructure-assisted perception by enabling semantic reasoning beyond raw sensor measurements [15]. Prior studies have explored lane-level mapping, trajectory-based topology inference, and map-assisted interpretation of vehicle motion for traffic monitoring and behavior analysis [14,15,21]. Infrastructure-assisted HD mapping frameworks have shown the capability to maintain accurate lane-level representations over time and to support behavior-level reasoning using vehicle trajectories collected from roadside sensors [15,21]. Such approaches are especially relevant to WWD detection, where the core challenge lies in determining whether observed vehicle motion is consistent with the intended lane direction and connectivity rather than simply detecting moving objects. Despite these advances, relatively few existing studies explicitly integrate real-time roadside perception, short-term trajectory prediction, and temporally validated lane-level reasoning within a framework specifically targeted at early WWD detection. This gap motivates the development of a direction-oriented, map-based reasoning framework tailored to complex ramp-terminal and access-zone environments, which is the focus of the present study.

## 3. System Overview

This section describes the overall structure of the proposed infrastructure-based WWD detection system, including its high-level architecture, roadside sensing configuration, and online processing workflow. The proposed framework is designed to detect potentially hazardous wrong-way maneuvers at complex access zones by combining roadside LiDAR sensing with lane-level semantic reasoning based on an HD map.

### 3.1. Overall System Architecture

**Figure 1:**
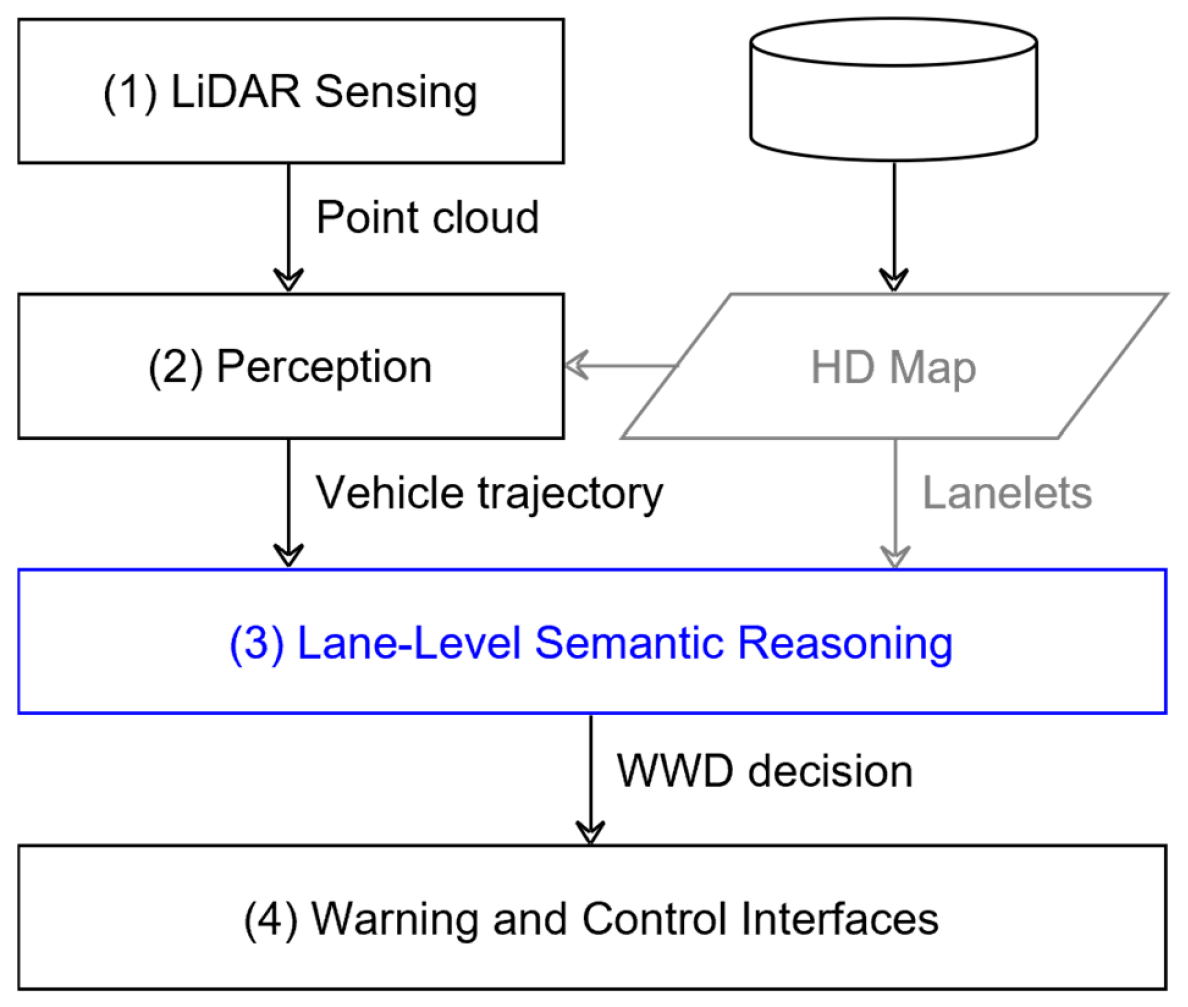
Overall architecture of the proposed LiDAR-based infrastructure system for WWD. Point-cloud observations from roadside LiDAR are processed through perception and combined with lanelet information derived from an HD map. The resulting map-assisted reasoning module determines the WWD state and forwards the decision to the warning and control interface.

The overall architecture of the proposed system is illustrated in Figure 1. The framework consists of four major functional stages: (1) LiDAR sensing, (2) perception, (3) lane-level semantic reasoning, and (4) warning and control interfaces. These components are sequentially connected to transform raw roadside observations into an event-level WWD decision. In the first stage, roadside LiDAR sensing provides three-dimensional point-cloud observations of vehicles approaching or moving through the monitored area. In the second stage, the perception module processes the raw point cloud to estimate vehicle-level motion states, including object position, identity continuity, and trajectory information. In parallel, lane-level information is obtained from the HD map and represented in the form of lanelets, which describe lane geometry, directionality, and connectivity. In the third stage, the perceived vehicle trajectory is evaluated together with the associated lanelet information through a map-assisted semantic reasoning process. This reasoning module determines whether the observed vehicle motion is consistent with the intended lane direction and whether the temporal evolution of the motion supports classification as a WWD event. Finally, the resulting WWD decision is delivered to the warning and control interface, which serves as the downstream output layer of the proposed framework. As shown in Figure 1, the key distinction of the proposed architecture lies in the integration of roadside LiDAR perception with lane-level semantic reasoning rather than reliance on raw trajectory information alone. This structure enables the system to interpret vehicle behavior in relation to the intended traffic flow of the mapped roadway environment.

### 3.2. Deployment and Roadside Configuration

The proposed system is designed for roadside deployment in geometrically complex traffic environments such as ramp terminals, access zones, and rest-area-related entrance regions. In such locations, wrong-way maneuvers may occur when a driver misinterprets roadway geometry, lane assignment, or entry direction under constrained visibility or high cognitive workload. To monitor these environments, a LiDAR sensor is installed at a fixed roadside location that provides sufficient visibility over the target area. The sensing position is selected to maximize coverage of the relevant vehicle approach paths while minimizing occlusion from surrounding infrastructure, roadside objects, or traffic itself. Because LiDAR directly measures three-dimensional spatial structure, it is suitable for capturing the position and motion of vehicles under both daytime and nighttime conditions. The roadside sensing configuration is complemented by an HD map of the monitored area. In the proposed framework, the HD map is not used merely as a background reference, but as a semantic representation of allowable traffic flow. Lanelets derived from the HD map encode lane boundaries, directionality, and topological connectivity, thereby providing the spatial basis for later behavioral reasoning. This combination of roadside sensing and lane-level map information forms the basis of the proposed infrastructure-centered WWD detection strategy.

### 3.3. Processing Pipeline

**Figure 2:**
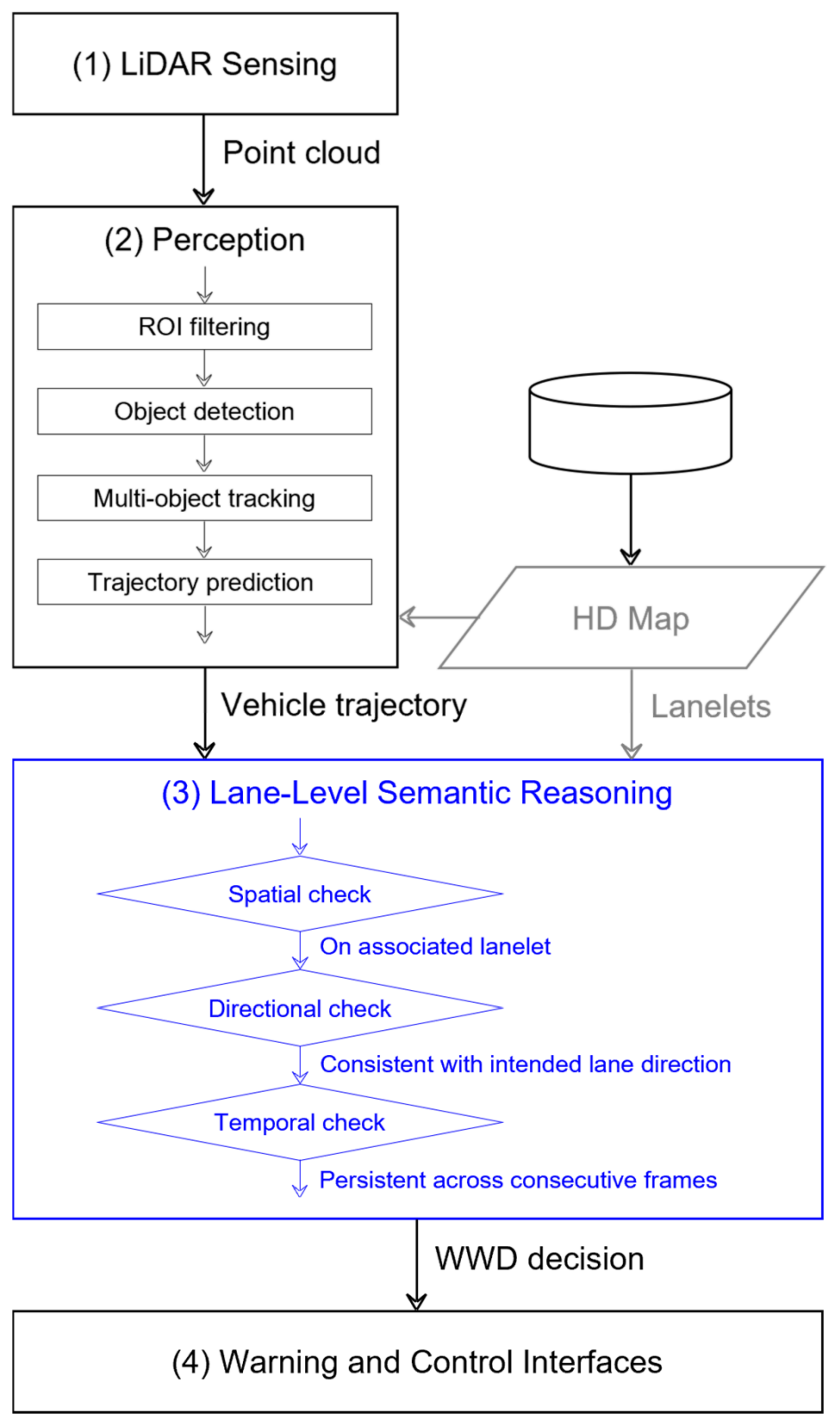
Online processing pipeline from roadside LiDAR sensing to event-level WWD detection. Raw point-cloud data are processed through filtering, vehicle detection, multi-object tracking, and short-term trajectory updating. The resulting vehicle trajectory is then evaluated using lanelet-based semantic reasoning, including spatial consistency, direction consistency, and temporal persistence checks, before a wrong-way event is confirmed.

The detailed online processing workflow of the proposed system is presented in Figure 2. Starting from roadside LiDAR point-cloud acquisition, the pipeline sequentially performs perception and semantic reasoning steps before generating an event-level WWD decision. Within the perception stage, the raw point cloud is first refined through region-of-interest (ROI) filtering so that irrelevant background structures and non-target regions are excluded from further processing. The filtered point cloud is then used for vehicle object detection, after which multi-object tracking is applied to maintain identity continuity across frames. Based on the tracked motion states, the system updates short-term vehicle trajectories and generates predicted paths, which provide motion information for the reasoning module. The lane-level semantic reasoning stage evaluates the resulting vehicle trajectory with respect to the corresponding lanelet information from the HD map. As shown in Figure 2, this reasoning process consists of three main checks: spatial consistency, direction consistency, and temporal persistence. The spatial check determines whether the vehicle is located within or appropriately associated with a valid lanelet region. The direction check evaluates whether the current vehicle heading and predicted-path direction are consistent with the intended lane direction of the associated lanelet. The temporal check verifies whether the detected inconsistency persists across consecutive observations rather than appearing as a short-lived transient artifact. Only when the required semantic and temporal conditions are satisfied is the observed motion confirmed as a WWD event.

The proposed WWD detector is implemented as a ROS 2 node. The upstream perception pipeline is implemented using Autoware Universe components for LiDAR CenterPoint detection, multi-object tracking, and map-based trajectory prediction [22,23,24]. It receives tracked object states and predicted paths through a PredictedObjects message interface and receives the Lanelet2-compatible vector map through an HADMapBin message interface. The map information is converted into a lanelet representation and used together with the received object states for semantic reasoning. The detector publishes the event-level WWD decision together with abnormal-object information, diagnostic data, visualization markers, and processing-time information through ROS 2 output interfaces. By structuring the workflow in this manner, the proposed system transforms roadside observations into an event-level safety decision while reducing the influence of isolated perception noise and transient directional inconsistencies.

### 3.4. Summary

In summary, the proposed system transforms roadside LiDAR observations into an event-level WWD decision through four sequential functions: LiDAR sensing, upstream perception and map-based prediction, lanelet-based semantic reasoning, and a downstream decision–output interface. The perception pipeline provides tracked object states and map-aware predicted paths, while the Lanelet2 HD map supplies the spatial and directional context required for semantic interpretation. The proposed WWD detector then combines lanelet association, lane-relative heading analysis, predicted-path direction verification, and consecutive-frame confirmation to generate an online decision. The following section describes the implementation details of the perception inputs and lanelet-based WWD decision logic.

## 4. Method

This section describes the core methodology of the proposed infrastructure-based WWD detection system. The framework combines roadside LiDAR perception with lane-level semantic reasoning based on an HD map in order to identify vehicle motion that is inconsistent with the intended lane direction. The overall procedure consists of LiDAR-based object detection, multi-object tracking, short-term trajectory prediction, HD map construction and lanelet representation, and lanelet-based WWD decision making.

### 4.1. LiDAR-Based Object Detection

The detection stage processes roadside LiDAR point clouds to identify vehicle objects within the monitored area. Because the sensor continuously observes the roadway in three dimensions, the resulting point-cloud data provide spatial measurements independent of ambient illumination conditions. To reduce unnecessary computation, the raw point cloud is first restricted to a predefined ROI corresponding to the roadway area relevant to WWD monitoring. Object-level observations are then extracted from the filtered point cloud to identify candidate vehicles.

The resulting detections provide the object-level spatial observations required for subsequent tracking. Sparse point returns, partial occlusion, and vehicle overlap may introduce uncertainty into the estimated position and orientation. The proposed WWD detector does not directly operate on raw point clouds or isolated detections; instead, it receives temporally associated object states and predicted paths generated by the upstream perception pipeline. Thus, the detection stage supplies the observations from which stable motion states can be estimated for downstream tracking, prediction, and lanelet-based reasoning.

In the implemented system, object detection is performed using the Autoware Universe LiDAR CenterPoint node (autoware_lidar_centerpoint), which processes the ROI-filtered roadside LiDAR point cloud to estimate vehicle position, size, orientation, and classification information [22]. The resulting three-dimensional detections are provided to the upstream multi-object tracking pipeline. Unless otherwise specified, the detection node uses the standard Autoware Universe configuration; the present study does not compare alternative detector architectures or optimize detector-specific parameters.

### 4.2. Multi-Object Tracking

The tracking stage maintains identity continuity for detected vehicles across consecutive frames. Tracking is essential because WWD recognition is based on temporally continuous motion rather than isolated instantaneous observations. For each detected object, the tracking stage associates observations belonging to the same physical vehicle and updates its motion state over time. The resulting tracked state includes object identity, position, velocity, and orientation.

The resulting tracked states provide an observed past-to-current motion history. This history supplies the current position, speed, and heading required for lanelet association and direction-consistency evaluation. The tracked states are subsequently provided to the map-based prediction stage, which extends the current state into a short-horizon future path hypothesis. Thus, tracking and prediction serve complementary roles: tracking estimates the observed motion history of a vehicle, whereas prediction provides a map-aware representation of its near-future motion for downstream WWD reasoning.

In the implemented system, multi-object tracking is performed using the Autoware Universe multi-object tracker node (autoware_multi_object_tracker) [23]. The tracker maintains object identity and kinematic states across consecutive frames through a Kalman-filter-based tracking framework. The tracked state provided to the downstream modules includes object identity, position, velocity, and orientation. Current detections are associated with existing tracks through the tracker’s gated data-association and assignment procedure. Unless otherwise specified, the tracker uses the standard Autoware Universe configuration; the present study does not separately optimize or compare tracker association parameters.

### 4.3. Short-Term Trajectory Prediction

The prediction stage generates short-term predicted paths for each active object based on the tracked motion state. This stage is not intended for long-horizon behavioral forecasting. Instead, it provides a map-aware near-future motion hypothesis using the tracked state and lane-level map information. When multiple predicted paths are available, the path with the highest confidence is selected for subsequent WWD reasoning.

The selected predicted path is used together with the current tracked state in the lanelet-based decision process. The current vehicle heading is first evaluated relative to the local direction of the matched lanelet. The directions of the midpoint and endpoint segments of the highest-confidence predicted path are then evaluated relative to the local direction of the same lanelet. A WWD candidate is generated only when the current heading and the selected predicted path both indicate directional inconsistency. Thus, prediction complements historical tracking by providing a map-aware future-motion representation used to verify whether the observed directional anomaly is likely to continue.

In the implemented system, short-term trajectory prediction is performed using the Autoware Universe map-based prediction node (autoware_map_based_prediction) [24]. The node receives tracked object states together with Lanelet2-compatible map information and generates multiple map-aware predicted-path hypotheses for active objects. For objects associated with mapped lanes, the prediction process considers lane-following and lane-change hypotheses using the tracked state and lane geometry. For objects in weakly mapped or unmapped regions, kinematic continuation models are used to generate feasible short-term paths. Each predicted path is assigned a confidence value, and the highest-confidence path is selected for downstream WWD reasoning. Unless otherwise specified, the prediction node uses the standard Autoware Universe configuration; the present study does not optimize the prediction horizon, number of hypotheses, or confidence-calibration parameters.

### 4.4. HD Map Construction and Lanelet Representation

**Figure 3:**
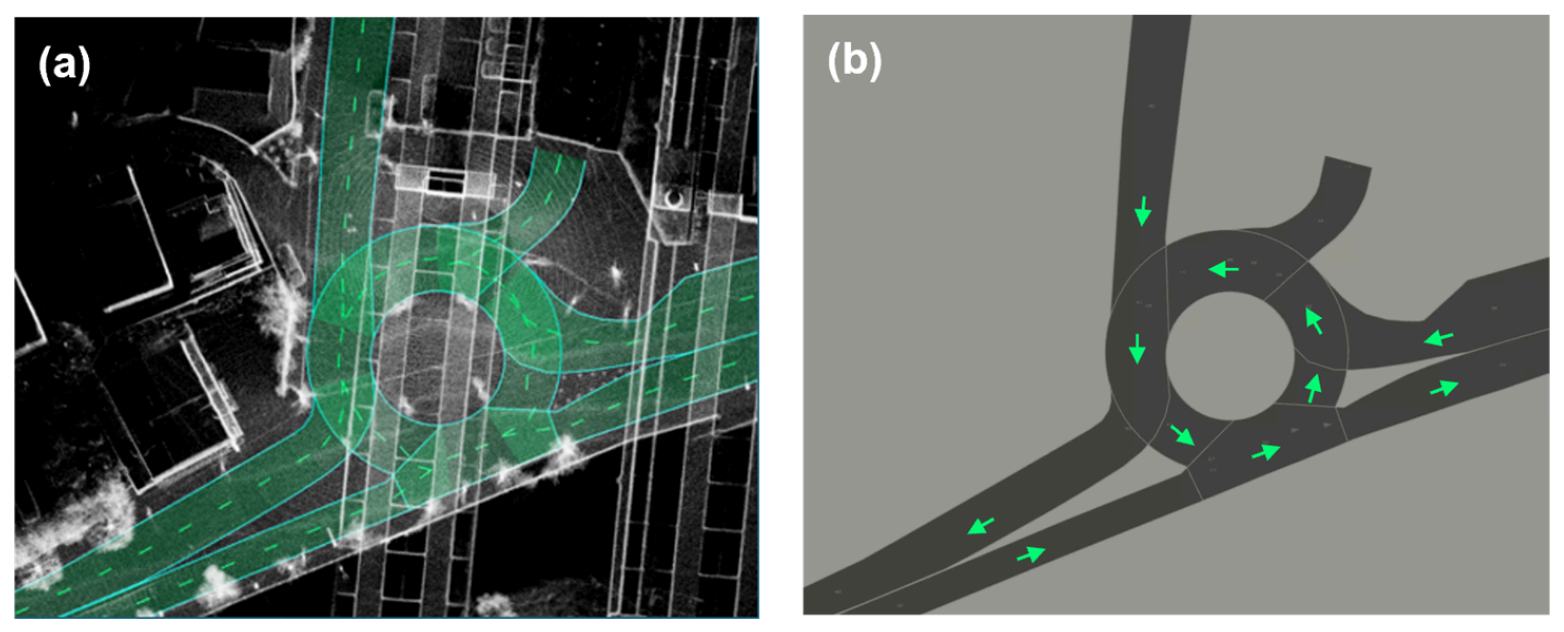
HD map composition and abstraction for lane-level semantic reasoning: (**a**) detailed HD map representation and (**b**) simplified lane-level representation derived for online reasoning. The HD map used in this study consists of detailed geometric and vectorized roadway information, from which a simplified lane-level representation is constructed for semantic reasoning. This abstraction provides the spatial and directional information required to associate tracked vehicles with valid roadway regions and to interpret their motion relative to the intended traffic flow.

**Figure 4:**
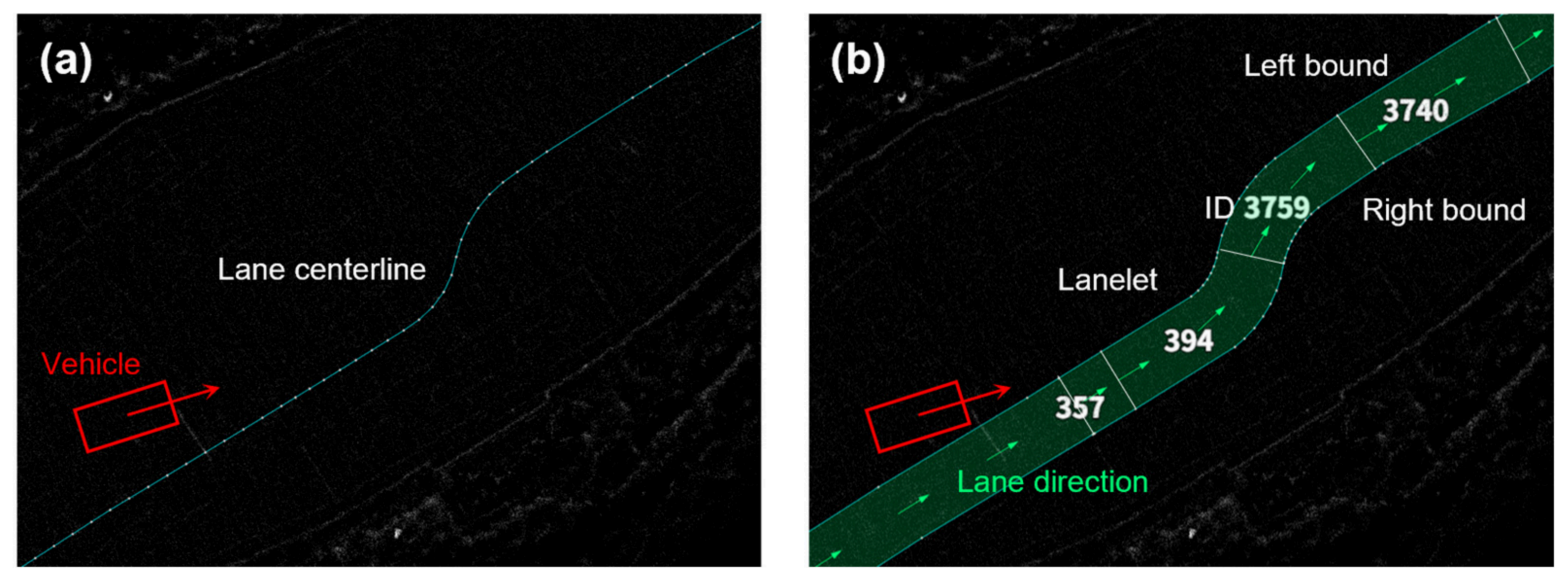
Comparison between (**a**) centerline-based representation and (**b**) lanelet-based drivable-area semantics. A centerline-only representation provides only a nominal reference path, whereas a lanelet explicitly represents the drivable area together with lane boundaries and directional semantics. The lanelet-based representation therefore provides stronger support for lane occupancy verification and wrong-way direction reasoning in complex roadway access environments.

In the proposed framework, the HD map serves as more than a geometric background. It provides lane-level semantic information, including roadway boundaries, intended lane directions, and topological relationships between adjacent road segments. As illustrated in Figure 3, the HD map used in this study can be understood as a combination of detailed roadway geometry and vectorized semantic road elements. From this richer map source, a simplified representation is derived for online reasoning so that the system can efficiently interpret vehicle motion in relation to the mapped traffic structure. The proposed method adopts a lanelet-based road representation for this purpose. A lanelet explicitly represents the drivable area of an individual lane segment together with its directional semantics. This is important because WWD detection requires not only knowledge of where a vehicle is located, but also whether its motion is consistent with the intended direction of travel for the occupied lane region. The rationale for adopting lanelets rather than a centerline-only representation is illustrated in Figure 4. A centerline provides only a nominal reference path and does not explicitly encode lane width or drivable-area occupancy. In contrast, a lanelet represents the full lane polygon together with its left and right boundaries, enabling direct verification of whether a tracked vehicle occupies a valid lane region. This representation also supports more reliable reasoning about directional consistency, especially in geometrically complex access zones where a centerline alone may be insufficient to distinguish correct from wrong-way motion. Accordingly, the HD map is transformed into a lanelet-based representation that can be directly used by the online reasoning stage. Each lanelet contains the geometric and directional information necessary for associating tracked vehicles with valid lane regions and for evaluating whether their headings are compatible with the mapped traffic flow.

### 4.5. Lanelet-Based Wrong-Way Driving Decision

Within the online processing pipeline shown in Figure 2, the WWD decision is made in the lanelet-based semantic reasoning stage. In this stage, each tracked vehicle is evaluated through three sequential checks: spatial consistency, direction consistency, and temporal persistence. This structure enables the system to transform tracked vehicle trajectories into event-level behavioral judgments while suppressing transient artifacts and isolated perception errors.

#### 4.5.1. Spatial Consistency Check

The first step of the decision process is to associate each detected object with a valid lanelet region. For each tracked object, its planar position is first tested against the polygonal area of each lanelet primitive using an occupancy-based inside test. If the object lies inside a lanelet, that lanelet is directly assigned to the object. If direct occupancy matching fails, nearby candidate lanelets are additionally examined as a boundary-aware fallback. In this secondary step, only lanelets located within a short distance from the object are considered, and the match is accepted only when the object remains sufficiently close to the lane boundary and its heading is reasonably aligned with the candidate lane direction. In the current implementation, this fallback requires the candidate lanelet to be within 3 m of the object and the angular difference to be less than 45°. Objects that fail both direct occupancy matching and boundary-aware fallback are excluded from wrong-way evaluation. This strategy improves robustness near lane boundaries and transitional roadway regions while suppressing false-positives (FPs) caused by objects that are not meaningfully associated with the drivable lane network.

#### 4.5.2. Direction Consistency Check

For objects successfully associated with a lanelet, wrong-way behavior is evaluated by comparing the object heading with the canonical direction of the matched lanelet. The object heading is represented as a unit vector formed from the tracked yaw angle, while the lanelet direction is represented by a unit vector corresponding to the intended traffic flow. The angular difference between the two directions is computed from the inner product of these unit vectors. A wrong-way condition is identified when this angular difference exceeds a predefined threshold. In the current implementation, the wrong-way threshold is set to 135°. In addition, wrong-way evaluation is performed only when the object speed exceeds a minimum threshold so that unstable heading estimates under near-stationary motion do not trigger erroneous detections. The current implementation uses a minimum speed threshold of 1 m/s for wrong-way evaluation. Because instantaneous heading estimates can fluctuate under low-speed motion, partial occlusion, or sparse point cloud observations, the implementation includes a heading stabilization step based on circular averaging. Instead of directly averaging scalar angles, heading vectors are aggregated in the unit-circle domain and then converted back to an angular representation. This approach avoids discontinuities at the 0°/360° boundary and reduces abrupt changes in heading caused by frame-to-frame noise. In the current implementation, this functionality is provided through the circular-mean option and is used to improve the robustness of heading-based anomaly detection.

#### 4.5.3. Temporal Persistence Check

To reduce false alarms caused by isolated perception errors or short-lived directional inconsistencies, the system confirms wrong-way behavior only after repeated detection over consecutive frames for the same tracked object. This temporal validation exploits the stable object identity maintained across frames and prevents single-frame heading outliers from generating immediate alerts. In the current implementation, a wrong-way state is confirmed only when the inconsistency persists for at least three consecutive frames. This validation rule provides a practical balance between early warning capability and decision reliability in real roadside sensing conditions.

**Figure 5:**
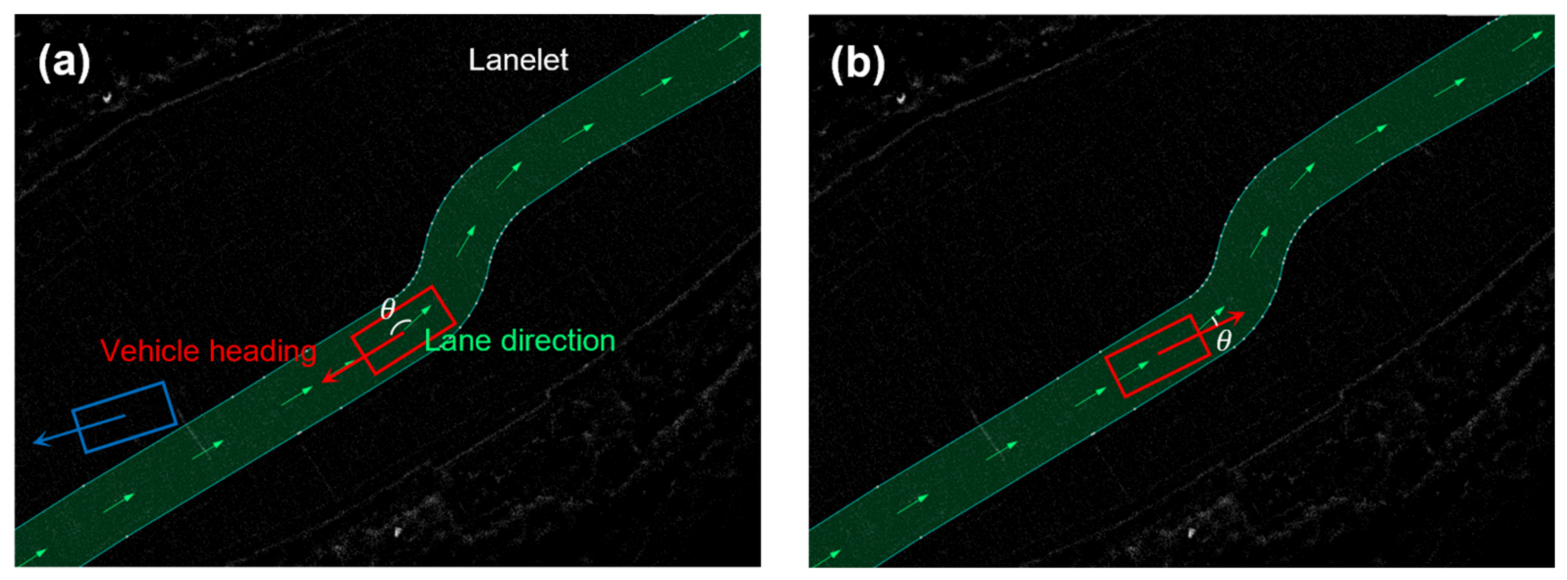
Lane-direction consistency analysis for WWD detection: (**a**) WWD with a large angular difference between the vehicle heading and the lane direction and (**b**) normal driving with a small angular difference. The heading vector of a tracked vehicle is compared with the intended direction of the associated lanelet and a wrong-way condition is identified when the angular difference exceeds the predefined threshold.

By jointly considering lanelet occupancy, heading-direction consistency, and temporal persistence, the proposed framework provides a semantically grounded and temporally stable basis for early WWD detection. In this way, the reasoning stage shown in Figure 2 is operationally realized through the directional comparison principle illustrated in Figure 5.

### 4.6. Summary

In summary, the proposed WWD decision method operates on tracked object states and map-based predicted paths expressed in the common map frame. The method first associates each object with a Lanelet2 region through polygon-based occupancy matching and a boundary-aware fallback procedure. It then evaluates the object’s current heading and the midpoint and endpoint segments of the highest-confidence predicted path relative to the local direction of the matched lanelet. A WWD candidate is generated only when both the current heading and the predicted path indicate directional inconsistency, and it is confirmed after three consecutive candidate detections. At the 10 Hz processing rate, three consecutive candidate frames span approximately 0.2 s from the first candidate timestamp to the third candidate timestamp, with the WWD confirmation generated at the third candidate frame.

## 5. Experimental Setup

This section describes the experimental environment, roadside sensing configuration, dataset composition, and evaluation methodology used to assess the proposed WWD detection system. Because the proposed framework is intended for deployment in complex access environments, the experiments were designed to evaluate both the practical sensing setup and the event-level detection performance under representative operating conditions.

### 5.1. Test Environment and Deployment Sites

**Figure 6:**
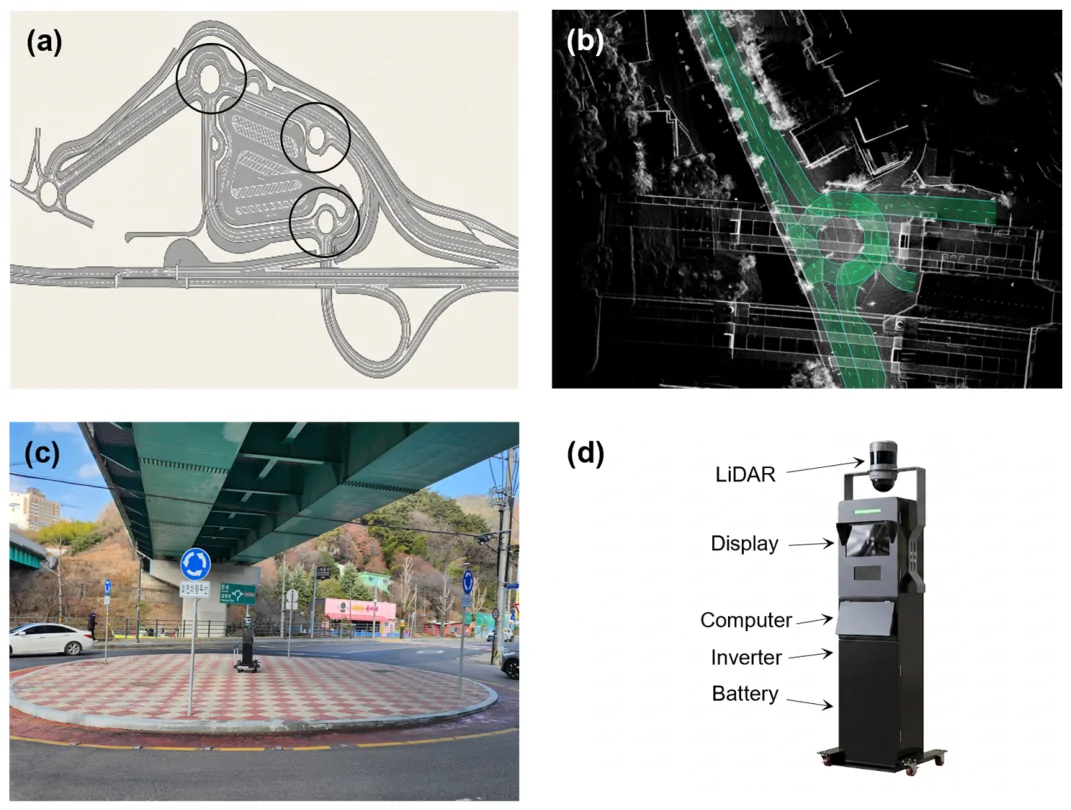
Target deployment site, surrogate evaluation site, and system configuration used for validation of the proposed WWD detection system: (**a**) target deployment site, with black circles indicating planned installation locations in complex roadway access environments; (**b**) HD map of the surrogate evaluation site; (**c**) photograph of the surrogate evaluation site; and (**d**) hardware configuration of the proposed system. These panels jointly present the intended deployment scenario, the surrogate validation environment, and the field-deployable sensing platform used in this study.

The proposed system was motivated by wrong-way risk at complex roadway access zones such as highway rest-area entrances. However, the intended highway rest-area deployment site was not available for full validation during the study period. Therefore, the event-level evaluation was conducted at a single surrogate roundabout site located at 108 Jiho-ro, Dong-gu, Gwangju, Republic of Korea. As shown in Figure 6, the experimental configuration distinguishes between the intended target deployment site and the surrogate evaluation site. The surrogate site was selected to evaluate the methodological challenges relevant to lane-level WWD reasoning, including curved lane geometry, multiple entry and exit approaches, short directional decision distances, and potential ambiguity in lanelet association. The site was not considered a geometrically or operationally equivalent replacement for a highway rest-area entrance. In particular, it differs from highway ramp and rest-area environments in lane configuration, traffic speed, geometric scale, traffic composition, and access-control characteristics. Accordingly, the present evaluation assesses the feasibility of the proposed lanelet-based WWD detection logic under the tested surrogate-site conditions. It does not establish system performance for all highway rest-area entrances, freeway ramps, or other roadway access geometries.

### 5.2. Sensor Configuration and Deployment

To monitor the evaluation site, a rotating three-dimensional LiDAR sensor was installed at a fixed roadside position with sufficient visibility over the area of interest. The system used a 40-channel Hesai Pandar40P LiDAR (Hesai Technology Co., Ltd., Shanghai, China), which was selected to provide dense three-dimensional observations of vehicle motion within the monitored region. The sensor was deployed at a height of approximately 1 m, and its position was chosen to maximize coverage of the relevant approach and access paths while minimizing occlusion from roadside structures and surrounding traffic. LiDAR data were processed on an on-site ASUS PB60G computer equipped with an Intel Core i5-9500T CPU and an NVIDIA GeForce GTX1650 GPU. The processing pipeline was implemented as a ROS 2-based system and included upstream object detection, multi-object tracking, map-based trajectory prediction, and the proposed lanelet-based WWD decision module. The LiDAR perception, prediction, and WWD decision pipeline operated at 10 Hz. The main sensor, computing, and deployment specifications are summarized in Table 1. The sensing setup is also illustrated in Figure 6, which presents the target site, the surrogate evaluation site, and the roadside deployment configuration together with the monitored coverage region. This figure is intended to clarify not only where the validation was performed, but also how the sensor was positioned to support continuous observation of vehicle trajectories in the access zone. Such placement is particularly important for WWD detection because reliable trajectory interpretation depends on maintaining visibility over the spatial region where incorrect entry or reverse-direction motion may first emerge.

To support lanelet-based reasoning, the roadside LiDAR observations and the Lanelet2 HD map were represented in a common local map frame. Because the LiDAR sensor was fixed throughout the experiment, its pose was estimated during system setup by normal-distributions transform (NDT) scan matching between the observed point cloud and a prebuilt point-cloud map. The resulting LiDAR-to-map transformation was then published as a static transform during operation and applied to the perception outputs before lanelet association and directional reasoning. The quality of the NDT alignment was monitored using the transform-probability and nearest-voxel transformation-likelihood metrics. These metrics were used to verify scan-to-map matching quality during system setup; a separate quantitative analysis of absolute registration error was not conducted in this study.

### 5.3. Dataset Composition and Annotation

The experimental dataset was collected under daytime and nighttime operating conditions to evaluate the proposed system under the two illumination conditions considered in this study. The recordings included both normal vehicle passes and staged WWD events within the monitored access region. The dataset was designed for event-level evaluation, in which each WWD case was represented as a temporal interval rather than as a collection of independently labeled frames. The composition of the validation dataset under each operating condition is summarized in Table 2.

Because naturally occurring WWD events are rare and intentionally inducing wrong-way maneuvers in uncontrolled public traffic would be unsafe, the WWD cases were collected through staged test drives in cooperation with the local police authority under partially controlled traffic conditions. Three test vehicles and three drivers participated in the data-collection procedure. These staged maneuvers were used to evaluate the event-level detection logic under controlled test conditions and were not intended to estimate the natural incidence or population frequency of WWD events.

The daytime recording lasted 14 min 41 s and contained 81 vehicle passes, including 14 annotated WWD events and 67 normal vehicle passes. The nighttime recording lasted 23 min 58 s and contained 110 vehicle passes, including 22 annotated WWD events and 88 normal vehicle passes. Overall, the dataset contained 191 vehicle passes, consisting of 36 annotated WWD events and 155 normal vehicle passes. A normal vehicle pass was defined as a passage through the monitored area that did not exhibit motion inconsistent with the intended direction of the associated lanelet. The WWD cases are reported as annotated event-level temporal intervals. Because the original data-collection record did not preserve an independent physical-maneuver identifier, the number of independently executed physical maneuvers was not separately recorded. Therefore, temporally adjacent event intervals associated with different tracker-generated Object IDs cannot be reliably classified retrospectively as separate physical maneuvers, repeated maneuvers, or tracker fragmentation. Accordingly, the reported events should not be interpreted as statistically independent maneuver trials.

Each WWD event was manually annotated as a temporal ground-truth (GT) interval. A WWD event was defined as a temporally continuous interval during which a tracked vehicle exhibited motion inconsistent with the intended direction of the associated lanelet. Accordingly, annotation focused on the presence, onset, and duration of sustained abnormal motion rather than on isolated frame-level anomalies. The GT intervals were annotated using the LiDAR perception visualization, which displayed the upstream detection, tracking, and map-based prediction results. The downstream WWD decision output was not displayed or used during annotation. The tracker-generated object ID was used only to associate each annotated interval with the corresponding tracked object in Appendix A Table A1 and Table A2; it was not used as a criterion for labeling vehicle motion as wrong-way or normal. Thus, the downstream decision output did not contribute to the labeling process. However, because the annotations were based on the upstream LiDAR perception visualization, they do not constitute sensor-independent GT. No camera recording or other independent reference source was collected or used for annotation in this study. Accordingly, the present evaluation should be interpreted as an assessment of the lanelet-based WWD reasoning module conditioned on the availability of upstream perception outputs, rather than as a fully independent end-to-end evaluation of missed detections, track losses, or identity-switch failures.

### 5.4. Evaluation Metrics

**Figure 7:**
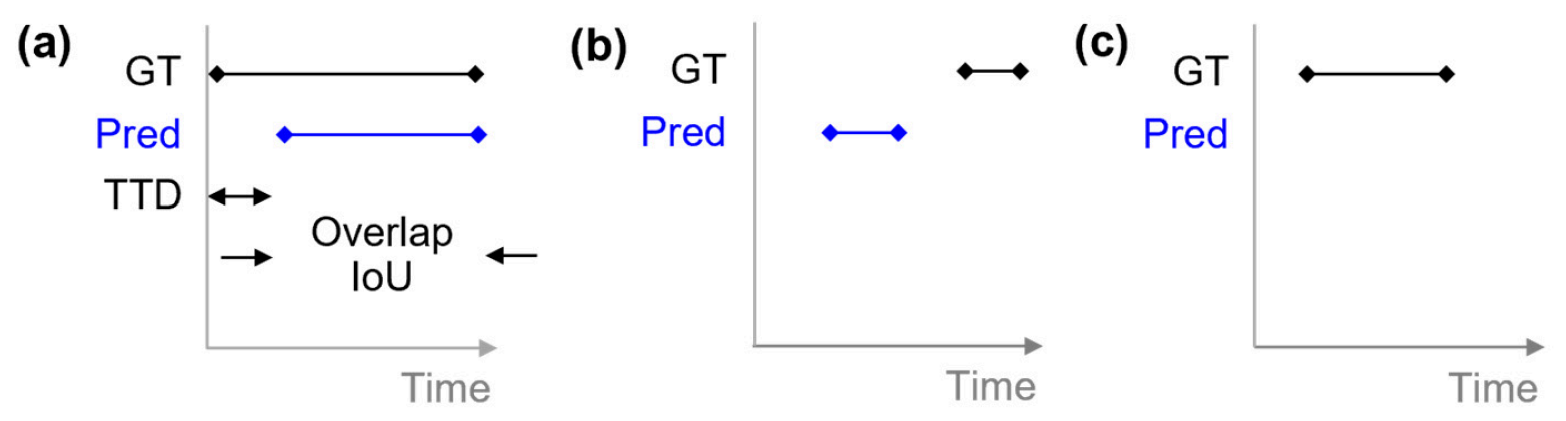
Event-level temporal correspondence used for offline evaluation of WWD detection: (**a**) a predicted event (Pred) matched to a GT interval is counted as a TP; (**b**) a predicted event without a matched GT interval is counted as an FP; (**c**) a GT interval without a matched prediction is counted as an FN. Event correspondence is determined when temporal IoU exceeds 0.1 or temporal overlap exceeds 0.3 s. TTD is defined as *t_pred_*_,*start*_ − *t_GT_*_,*start*_. These criteria are used only for offline evaluation and are not part of the online WWD decision logic.

The proposed system was evaluated at the event level rather than the frame level. GT and predicted WWD events were represented as temporal intervals. As illustrated in Figure 7, a predicted event was counted as a true-positive (TP) when it was matched to a GT event according to a predefined temporal correspondence rule. A predicted event without a matched GT event was counted as an FP, whereas a GT event without a matched prediction was counted as a false negative (FN). Event correspondence was determined when either the temporal intersection-over-union (IoU) exceeded 0.1 or the temporal overlap duration exceeded 0.3 s. These values were used only for offline event matching and were not used by the online WWD decision algorithm. Event matching was performed on a one-to-one basis, such that each predicted event was matched to at most one GT event and each GT event was matched to at most one predicted event. The correspondence rule was selected to accommodate early event recognition, for which a system-generated event may begin before the manually annotated event onset and may not coincide exactly with the annotated temporal boundary. Accordingly, the resulting detection rate represents event-level matching performance under the specified temporal correspondence rule and should not be interpreted as a strict temporal-boundary localization metric. For matched events, the time-to-detection (TTD) was defined as

*TTD* = *t_pred_*_,*start*_ − *t_GT_*_,*start*_,

where *t_pred_*_,*start*_ is the start time of the system-predicted event and *t_GT_*_,*start*_ is the start time of the manually annotated event. A negative TTD indicates that the predicted event interval began earlier than the annotated interval. Because manual event-boundary annotation is subject to temporal uncertainty, TTD should be interpreted as a relative event-onset timing measure rather than direct evidence of end-to-end warning lead time or prevention effectiveness. Based on these definitions, the main evaluation metrics were the Detection Rate (DR), False Alarm Rate (FAR), and Average Time to Detection (Avg TTD). DR measures the proportion of GT WWD events that were matched under the specified correspondence rule, while FAR quantifies the proportion of normal instances that were incorrectly classified as WWD events. Avg TTD summarizes the relative onset timing over matched events.

## 6. Results and Discussion

**Figure 8:**
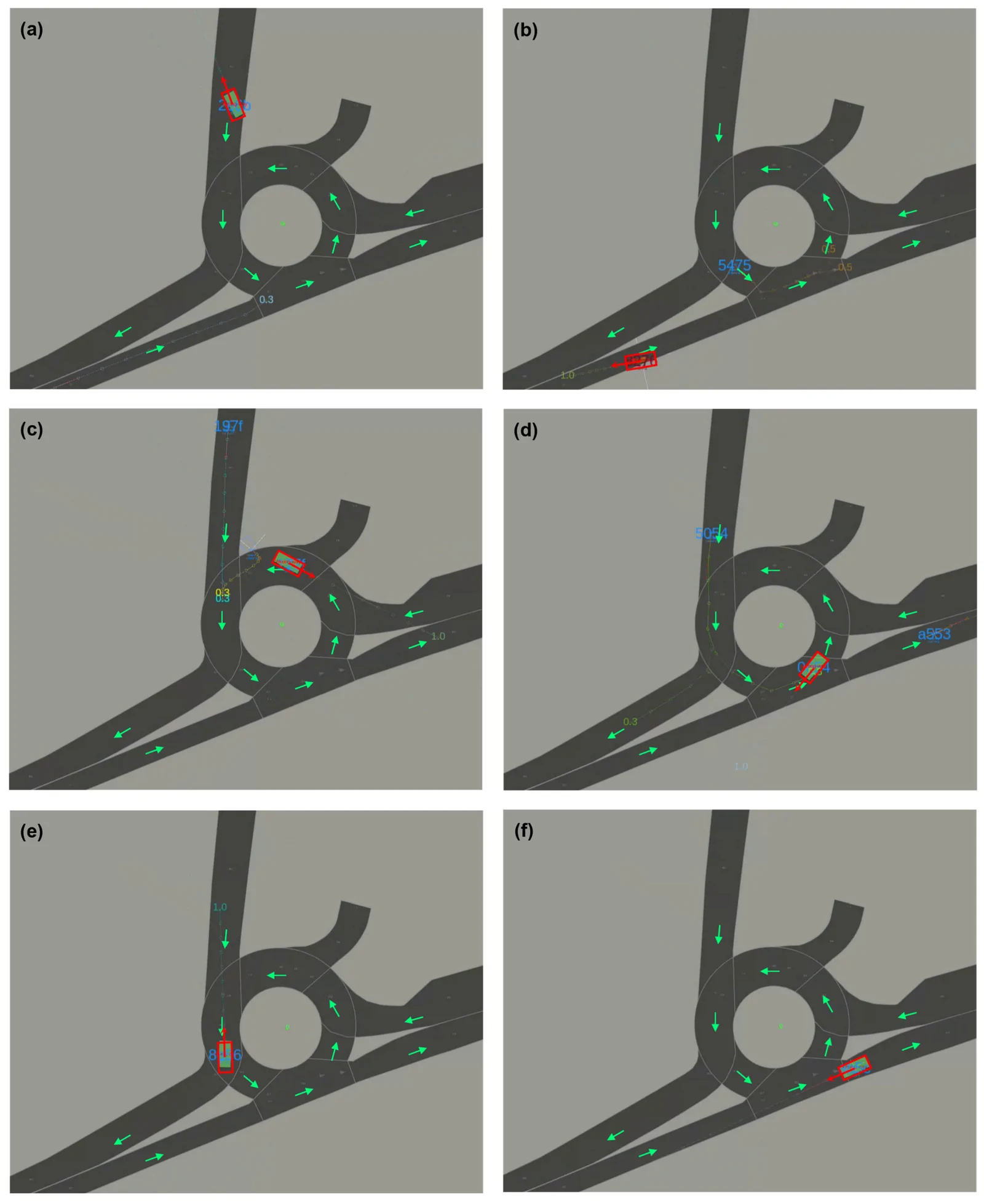
Graphical examples of WWD detection under daytime and nighttime conditions, with (**a**–**c**) showing representative daytime cases and (**d**–**f**) showing representative nighttime cases. Each panel presents a lanelet-based bird’s-eye-view overlay showing the tracked vehicle state, heading direction, and WWD event indication. These examples illustrate how the proposed framework identifies vehicle motion (red) that is inconsistent with the intended lane direction (green). Detailed event-level matching results are provided in Appendix A Table A1 and Table A2.

This section presents the event-level performance of the proposed WWD detection system under daytime and nighttime validation conditions. Following the evaluation framework described in the previous section, and using the method and evaluation parameter settings summarized in Table 3, performance was assessed using the numbers of TP, FP, and FN, together with the DR, FAR, and Avg TTD. The overall performance under the two validation conditions is summarized in Table 4, representative graphical detection cases are presented in Figure 8, and detailed per-event matching results are provided in Appendix A Table A1 and Table A2.

To examine the sensitivity of the event-level results to the offline temporal correspondence criteria, Table 5 summarizes matching performance under alternative IoU- and overlap-based thresholds. Under the original rule of temporal IoU > 0.1 or temporal overlap > 0.3 s, all 36 annotated GT events were matched. Because all matched events also satisfied temporal IoU > 0.1, the IoU-only criterion at this threshold likewise produced a 100.0% detection rate. Under more stringent criteria, the matched-event rate decreased to 86.1% for temporal IoU > 0.3, 77.8% for temporal IoU > 0.5, and 88.9% for temporal overlap > 0.5 s. These results indicate that the reported 100.0% detection rate is specific to the stated event-correspondence protocol and should not be interpreted as strict temporal-boundary localization performance.

### 6.1. Daytime Validation

In the daytime validation, all 14 annotated GT WWD events were matched by predicted events under the specified temporal correspondence rule, with no FNs, as summarized in Table 4. No FP event was observed among the 67 normal vehicle passes. The Avg TTD was −0.36 s, indicating that the system-generated event intervals generally began earlier than the manually annotated GT intervals under the specified temporal correspondence rule. These results represent event-level detection performance for the evaluated daytime validation data and should not be interpreted as a long-term false-alarm guarantee. Representative daytime examples are shown in Figure 8a–c. The lanelet-based bird’s-eye-view overlays illustrate the tracked vehicle state, vehicle heading, lane direction, and WWD event indication for representative daytime cases.

### 6.2. Nighttime Validation

In the nighttime validation, all 22 annotated GT WWD events were matched by predicted events under the specified temporal correspondence rule, with no FNs, as reported in Table 4. No FP event was observed among the 88 normal vehicle passes. The Avg TTD was −0.297 s, indicating that the system-generated event intervals generally began earlier than the manually annotated GT intervals under the specified temporal correspondence rule. These results represent event-level detection performance for the evaluated nighttime validation data and do not establish long-duration or deployment-wide operational reliability. Representative nighttime examples are shown in Figure 8d–f. The lanelet-based bird’s-eye-view overlays illustrate the tracked vehicle state, vehicle heading, lane direction, and WWD event indication for representative nighttime cases.

### 6.3. Discussion

#### 6.3.1. Interpretation of Event-Level Results

The results summarized in Table 4 indicate that the proposed system achieved a 100% event-level detection rate under the specified temporal correspondence rule in both daytime and nighttime validation conditions. Detailed per-event matching results in Appendix A Table A1 and Table A2 show the temporal relationships between the system-generated and manually annotated event intervals in terms of temporal overlap, IoU, and TTD. The graphical cases in Figure 8 further illustrate how lanelet occupancy, direction inconsistency, and temporal persistence were jointly represented in the evaluated scenes. The event-level evaluation was designed to assess whether the proposed framework generated a WWD decision during, or sufficiently close to the onset of, the manually annotated hazardous-motion interval. The temporal IoU and overlap criteria were therefore used as event-correspondence rules rather than strict temporal-localization thresholds. Accordingly, the reported detection rate should be interpreted under the stated matching protocol. Similarly, negative TTD values indicate earlier system-event onset relative to manual annotation and do not, by themselves, quantify end-to-end warning lead time, prevention effectiveness, or downstream warning performance. They depend on the event-onset definition, temporal annotation uncertainty, the 10 Hz update interval, and the absence of downstream warning-actuation latency in the evaluation.

#### 6.3.2. Evaluation Scope and Limitations

The present results should first be interpreted within the scope of a staged, single-site evaluation. The WWD events were generated through controlled test drives involving three test vehicles and three drivers, rather than collected as naturally occurring events during long-term traffic operation. The evaluation was conducted at one surrogate roundabout site and did not include direct validation at highway rest-area entrances, freeway ramps, grade-separated interchanges, or multi-lane access facilities. The recordings covered daytime and nighttime illumination conditions but did not include rain, snow, fog, severe weather, dense traffic, or prolonged occlusion. Therefore, the results demonstrate feasibility under the evaluated surrogate-site conditions but do not establish deployment-wide performance across roadway geometries, traffic conditions, or environments.

No FP event was observed among the 67 daytime and 88 nighttime normal vehicle passes. This corresponds to zero observed FP events during 14 min 41 s of daytime recording and 23 min 58 s of nighttime recording. However, the observation duration and number of normal passes are insufficient to establish a deployment-wide false-alarm rate or long-term operational reliability. In addition, the present validation did not exhaustively include legal or non-hazardous maneuvers that may temporarily resemble directional inconsistency, such as lane changes, boundary transitions, reversing, U-turn-like movements, tight turns, low-speed maneuvers, temporary stopping, or complex interactions among multiple vehicles. Accordingly, the absence of observed FP events in the present dataset does not establish false-alarm performance for these untested conditions.

The interpretation should also consider uncertainty in the upstream perception pipeline. Sparse point returns, partial occlusion, vehicle overlap, and tracking discontinuities may affect estimated object position and orientation and, consequently, lanelet association and predicted-path consistency. The implemented decision logic is intended to reduce the influence of short-lived fluctuations through speed gating, circular heading averaging, predicted-path direction verification, and consecutive-frame confirmation. However, these mechanisms do not eliminate the effects of missed detections, prolonged track loss, or identity switches. Such upstream failures may delay or prevent event confirmation even when the decision logic itself is functioning as designed.

The threshold values used in this study were selected for the present surrogate site and 10 Hz roadside LiDAR configuration. Although their functional roles are described explicitly, the present evaluation does not establish their global optimality or transferability to different lane geometries, sensor placements, traffic densities, weather conditions, or map-registration errors. In addition, the present study did not include a quantitative ablation analysis to isolate the individual contributions of lanelet association, predicted-path verification, circular heading averaging, or temporal persistence. Consequently, the observed detection and FP results cannot be attributed to any single component of the proposed decision logic or separated from the characteristics of the evaluated staged cases. Accordingly, the present study does not make a component-wise effectiveness claim for lanelet association, predicted-path verification, circular heading averaging, or temporal persistence. Comparative evaluation against a centerline-based association variant and versions without predicted-path verification, circular heading averaging, or temporal persistence remains future work.

#### 6.3.3. Future Validation Directions

Future work will extend the evaluation through multi-site, multi-season, and long-duration monitoring with naturalistic traffic data. Such studies should include highway rest-area entrances, freeway ramps, grade-separated interchanges, multi-lane access facilities, and diverse traffic densities and weather conditions. They should also include legal maneuvers that can resemble directional inconsistency and report FP events per hour over substantially longer observation periods. Comparative evaluation against centerline-based association, no-prediction-verification, no-circular-mean, and no-temporal-validation variants is needed to quantify the contribution of each design component. Future deployment studies should further evaluate long-duration runtime behavior, sensor uptime, and end-to-end latency from sensor acquisition to downstream warning delivery. These extensions would provide stronger evidence regarding operational reliability, component-wise effectiveness, and the practical value of the proposed framework beyond the present staged surrogate-site evaluation.

## 7. Conclusions

This study proposed a LiDAR-based infrastructure system for the early detection of WWD in complex access environments. By combining roadside LiDAR sensing with HD-map-based lanelet semantics, the proposed framework evaluates vehicle motion at the event level through lane occupancy, direction consistency, and temporal persistence. Experimental validation under both daytime and nighttime conditions matched all annotated WWD events under the specified event-level correspondence rule, and no FP event was observed among the normal vehicle passes included in the evaluated cases. The results support the feasibility of lanelet-based, roadside LiDAR WWD detection under the evaluated staged surrogate-site conditions. They do not establish deployment-wide performance, long-term operational stability, end-to-end warning latency, or direct prevention effectiveness. Future work will evaluate the framework at multiple roadway access geometries, including highway ramps and rest-area entrances, under diverse traffic, weather, and long-duration operating conditions.

## Figures and Tables

**Table 1:** System and deployment specifications used for field validation of the proposed WWD detection system.

Category	Item	Specification
Deployment site	Validation site	108 Jiho-ro, Dong-gu, Gwangju, Republic of Korea
	Site role	Surrogate evaluation site
	Roundabout outer diameter	22 m Approx.
	Number of circulating lanes	1
	Number of entry approaches	3
	Number of exit approaches	2
Sensing hardware	LiDAR model	Hesai Pandar40P
	Channels	40
	Field of view	360° (H), −25–15° (V)
	Mounting height	1 m
	Deployment type	Fixed installation at the central island of the roundabout
Computing hardware	Computer model	ASUS PB60G
	CPU	Intel Core i5-9500T
	GPU	NVIDIA GeForce GTX1650
Processing configuration	Processing framework	ROS 2-based pipeline
	Pipeline rate	10 Hz
Map alignment	Coordinate frame	Common local map frame
	Alignment method	NDT scan matching against a prebuilt PCD map
	Runtime transform	Static LiDAR-to-map TF
Validation setup	Operating conditions	Daytime/Nighttime

**Table 2:** Composition of the staged validation dataset under each operating condition.

Condition	Duration	Vehicle Passes	Annotated WWD Events	Normal Vehicle Passes
Daytime	14 min 41 s	81	14	67
Nighttime	23 min 58 s	110	22	88
Overall	38 min 39 s	191	36	155

**Table 3:** Method and evaluation parameters used in this study.

Category	Section	Parameter (Threshold)	Value	Purpose
Method	Section 4.5.1	Fallback distance	3 m	Candidate lanelets are additionally examined only when they are within this distance from the object.
		Fallback heading	45°	Fallback association is accepted only when the object heading is reasonably aligned with the candidate lane direction.
	Section 4.5.2	Wrong-way angle	135°	A wrong-way condition is identified when the angular difference between the object heading and the lanelet direction exceeds this threshold.
		Minimum speed	1 m/s	Wrong-way evaluation is performed only when the object speed exceeds this threshold.
		Heading stabilization	Circular mean	Heading vectors are aggregated in the unit-circle domain to reduce heading fluctuation and avoid 0°/360° discontinuity.
	Section 4.5.3	Persistence	3 frames	A wrong-way state is confirmed only when the inconsistency persists for at least three consecutive frames.
Evaluation	Section 5.4	Temporal IoU	0.1	A predicted event is considered matched when the temporal IoU exceeds this threshold or the overlap duration exceeds the minimum overlap threshold.
		Minimum overlap	0.3 s	

**Table 4:** Summary of event-level WWD detection performance under the specified offline temporal correspondence rule. FAR denotes the observed fraction of normal vehicle passes for which an FP WWD event was generated. The observed FP event rates are calculated over the stated recording durations and do not represent deployment-wide false-alarm performance.

Condition	GT Events	Pred Events	TP	FP	FN	DR (%)	FAR (%)	Normal Vehicle Passes	Observed FP Events/h	Avg TTD (s)
Daytime	14	14	14	0	0	100	0	67	0	−0.36
Nighttime	22	22	22	0	0	100	0	88	0	−0.297
Overall	36	36	36	0	0	100	0	155	0	−0.322

**Table 5:** Sensitivity of event-level matching performance to alternative offline temporal correspondence criteria. The original evaluation rule was temporal IoU > 0.1 or temporal overlap > 0.3 s. The alternative criteria are reported for sensitivity analysis only and do not alter the online WWD decision logic.

Matching Criterion	Matched GT Events/36	Detection Rate (%)
Original rule	36	100
Temporal IoU > 0.1 only	36	100
Temporal overlap > 0.3 s only	34	94.4
Temporal IoU > 0.3 only	31	86.1
Temporal overlap > 0.5 s only	32	88.9
Temporal IoU > 0.5 only	28	77.8

## Data Availability

The data presented in this study are not publicly available due to privacy, safety, and site-related restrictions. Limited information may be made available by the corresponding author upon reasonable request and subject to applicable permissions.

## Abbreviations

The following abbreviations are used in this manuscript:

WWD	Wrong way driving
LiDAR	Light detection and ranging
HD	High definition
ROI	Region of interest
IoU	Intersection over union
TTD	Time to detection
TP	True positive
FP	False positive
FN	False negative
DR	Detection rate
FAR	False alarm rate

## Appendix A

**Table A1:** Detailed event-level matching results for daytime validation.

No.	Object ID	GT Start Time (s)	GT End Time (s)	Pred Start Time (s)	Pred End Time (s)	TTD (s)	Overlap (s)	IoU	Representative Examples Used in Figure 8
1	f263	2559.868	2564.119	2559.018	2563.618	−0.85	3.75	0.735	
2	9065	2600.47	2617.794	2600.419	2617.519	−0.051	17.049	0.981	
3	2d6b	2754.447	2759.32	2754.321	2759.32	−0.126	4.873	0.975	(a)
4	4f74	2988.435	2989.489	2988.224	2988.623	−0.211	0.188	0.149	
5	5475	3030.199	3031.349	3030.124	3030.525	−0.075	0.326	0.266	
6	5e76	3033.686	3034.385	3033.725	3033.924	0.039	0.199	0.285	
7	8276	3034.763	3040.44	3034.328	3039.927	−0.435	5.164	0.845	(b)
8	2078	3078.176	3083.274	3077.824	3083.024	−0.352	4.848	0.89	
9	437a	3131.151	3141.2	3130.325	3140.401	−0.826	9.25	0.851	
10	2e7c	3179.476	3184.764	3179.601	3183.601	0.125	4	0.756	
11	5e7c	3187.257	3193.876	3186.101	3188.301	−1.156	1.044	0.134	
12	fe7d	3226.201	3236.289	3225.901	3234.801	−0.3	8.6	0.828	
13	897e	3241.576	3243.727	3241.301	3243.201	−0.275	1.625	0.67	
14	167f	3254.547	3264.3	3254.002	3263.402	−0.545	8.855	0.86	(c)

**Table A2:** Detailed event-level matching results for nighttime validation.

No.	Object ID	GT Start Time (s)	GT End Time (s)	Pred Start Time (s)	Pred End Time (s)	TTD (s)	Overlap (s)	IoU	Representative Examples Used in Figure 8
1	1346	8349.907	8350.409	8349.301	8350.223	−0.606	0.316	0.285	
2	6648	8408.558	8410.133	8408.124	8409.401	−0.434	0.843	0.419	
3	1253	8680.913	8688.836	8680.703	8688.005	−0.21	7.092	0.872	
4	0654	8714.264	8719.12	8714.004	8718.802	−0.26	4.538	0.887	(d)
5	2056	8759.133	8763.437	8759.005	8763.21	−0.128	4.077	0.92	
6	8e56	8770.763	8779.837	8769.903	8779.403	−0.86	8.64	0.87	(e)
7	845b	8897.488	8902.589	8896.905	8901.705	−0.583	4.217	0.742	
8	965c	8925.114	8930.54	8924.409	8928.605	−0.705	3.491	0.569	
9	aa5c	8929.654	8932.714	8928.405	8931.708	−1.249	2.054	0.477	(f)
10	f85e	8986.215	8996.615	8985.406	8996.706	−0.809	10.4	0.92	
11	5f61	9054.596	9057.167	9055.307	9056.809	0.711	1.502	0.584	
12	5364	9132.143	9136.142	9131.708	9135.407	−0.435	3.264	0.736	
13	7965	9163.943	9164.967	9164.008	9166.908	0.065	0.959	0.323	
14	f865	9164.967	9170.418	9164.51	9169.808	−0.457	4.841	0.819	
15	8068	9229.993	9234.793	9229.408	9234.609	−0.585	4.616	0.857	
16	0569	9273.519	9277.119	9273.41	9276.509	−0.109	2.99	0.806	
17	ca6c	9341.27	9344.346	9341.314	9343.213	0.044	1.899	0.617	
18	0f6f	9397.395	9409.978	9397.31	9408.811	−0.085	11.416	0.901	
19	1972	9475.298	9480.249	9475.012	9480.212	−0.286	4.914	0.938	
20	7973	9510.347	9514.971	9510.315	9513.114	−0.032	2.767	0.594	
21	8a76	9588.798	9596.1	9588.713	9595.515	−0.085	6.717	0.909	
22	4377	9611.948	9614.623	9612.513	9613.913	0.565	1.4	0.523	

## References

[1] National Transportation Safety Board (2012). Wrong-Way Driving.

[2] AAA Foundation for Traffic Safety (2021). Fatal Wrong-Way Crashes on Divided Highways.

[3] Cooner S.A., Cothron A.S., Ranft S.E. (2004). Countermeasures for Wrong-Way Movement on Freeways: Overview of Project Activities and Findings.

[4] Iowa State University Institute for Transportation (2018). Investigation of Wrong-Way Driving.

[5] Zhou H., Zhao J., Fries R., Gahrooei M.R., Wang L., Vaughn B., Bahaaldin K., Ayyalasomayajula B. (2012). Investigation of Contributing Factors Regarding Wrong-Way Driving on Freeways.

[6] Richard C.M., Lichty M.G. (2013). Driver Expectations When Navigating Complex Interchanges.

[7] Finley M.D., Balke K.N., Rajbhandari R., Chrysler S.T., Dobrovolny C.S., Trout N.D., Avery P., Vickers D., Mott C. (2016). Conceptual Design of a Connected Vehicle Wrong-Way Driving Detection and Management System.

[8] Iowa State University Institute for Transportation (2020). Automating Wrong-Way Driving Detection Using Existing CCTV Cameras.

[9] Creß C., Bing Z., Knoll A.C. (2024). Intelligent Transportation Systems Using Roadside Infrastructure: A Literature Survey. IEEE Trans. Intell. Transp. Syst..

[10] Finley M.D., Venglar S.P., Florence D., Charara H., Brydia R.E., Brewer M.A. (2024). Evaluation of Wrong-Way Driving Detection Technologies.

[11] Liang J., Yang K., Tan C., Wang J., Yin G. (2025). Enhancing high-speed cruising performance of autonomous vehicles through integrated deep reinforcement learning framework. IEEE Trans. Intell. Transp. Syst..

[12] Liang J., Tan C., Yan L., Zhou J., Yin G., Yang K. (2025). Interaction-aware trajectory prediction for safe motion planning in autonomous driving: A transformer-transfer learning approach. IEEE Trans. Intell. Transp. Syst..

[13] Zhao J., Xu H., Tian Y., Liu H. (2022). Towards Application of LiDAR Sensor to Traffic Detection: An Investigation of Its Built-In Features and Installation Techniques.

[14] Elghazaly G., Frank R., Harvey S., Safko S. (2023). High-definition maps: Comprehensive survey, challenges, and future perspectives. IEEE Open J. Intell. Transp. Syst..

[15] He Y., Bian C., Xia J., Shi S., Yan Z., Song Q., Xing G. VI-Map infrastructure-assisted real-time HD mapping for autonomous driving. Proceedings of the ACM MobiCom ’23: 29th Annual International Conference on Mobile Computing and Networking.

[16] Zhang T., Jin P.J. (2022). Roadside LiDAR vehicle detection and tracking using range and intensity background subtraction. J. Adv. Transp..

[17] Zhang J., Xiao W., Coifman B., Mills J.P. (2020). Vehicle tracking and speed estimation from roadside lidar. IEEE J. Sel. Top. Appl. Earth Obs. Remote Sens..

[18] Zhang Y., Luo Q., Zhang S., Liu H., Xie S. (2025). Anchor-free real-time vehicle tracking using LiDAR data. PLoS ONE.

[19] Ge Y., Jin P.J., Zhang T.T., Chen A. (2024). Roadside LiDAR sensor configuration assessment and optimization methods for vehicle detection and tracking in connected and automated vehicle applications. Transp. Res. Rec..

[20] Luo Q., Xu Z., Zhang Y., Igene M., Bataineh T., Soltanirad M., Jimee K., Liu H. (2025). Vehicle trajectory repair under full occlusion and limited datapoints with roadside LiDAR. Sensors.

[21] Liu H., Lin C., Gong B., Wu D. (2023). Automatic lane-level intersection map generation using low-channel roadside LiDAR. IEEE/CAA J. Autom. Sin..

[22] Autoware Foundation Autoware LiDAR CenterPoint. https://autowarefoundation.github.io/autoware_universe/main/perception/autoware_lidar_centerpoint/.

[23] Autoware Foundation Autoware Multi-Object Tracker. https://autowarefoundation.github.io/autoware_universe/main/perception/autoware_multi_object_tracker/.

[24] Autoware Foundation Autoware Map-Based Prediction. https://autowarefoundation.github.io/autoware_universe/main/perception/autoware_map_based_prediction/.

